# The Protective Role of Sestrin2 in Atherosclerotic and Cardiac Diseases

**DOI:** 10.3390/ijms22031200

**Published:** 2021-01-26

**Authors:** Yoshimi Kishimoto, Kazuo Kondo, Yukihiko Momiyama

**Affiliations:** 1 Department of Food Science and Human Nutrition, Faculty of Agriculture, Setsunan University, 45-1 Nagaotouge-cho, Hirakata, Osaka 573-0101, Japan; 2 Ochanomizu University, 2-1-1 Otsuka, Bunkyo-ku, Tokyo 112-8610, Japan; k24kondo523@gmail.com; 3 Department of Cardiology, National Hospital Organization Tokyo Medical Center, 2-5-1 Higashigaoka, Meguro-ku, Tokyo 152-8902, Japan; ymomiyamajp@gmail.com

**Keywords:** atherosclerosis, carotid plaque, coronary artery disease, inflammation, oxidative stress, sestrin2

## Abstract

Atherosclerotic disease, such as coronary artery disease (CAD), is known to be a chronic inflammatory disease, as well as an age-related disease. Excessive oxidative stress produced by reactive oxygen species (ROS) contributes to the pathogenesis of atherosclerosis. Sestrin2 is an anti-oxidant protein that is induced by various stresses such as hypoxia, DNA damage, and oxidative stress. Sestrin2 is also suggested to be associated with aging. Sestrin2 is expressed and secreted mainly by macrophages, endothelial cells, and cardiomyocytes. Sestrin2 plays an important role in suppressing the production and accumulation of ROS, thus protecting cells from oxidative damage. Since sestrin2 is reported to have anti-oxidant and anti-inflammatory properties, it may play a protective role against the progression of atherosclerosis and may be a potential therapeutic target for the amelioration of atherosclerosis. Regarding the association between blood sestrin2 levels and atherosclerotic disease, the blood sestrin2 levels in patients with CAD or carotid atherosclerosis were reported to be high. High blood sestrin2 levels in patients with such atherosclerotic disease may reflect a compensatory response to increased oxidative stress and may help protect against the progression of atherosclerosis. This review describes the protective role of sestrin2 against the progression of atherosclerotic and cardiac diseases.

## 1. Introduction

Cardiovascular diseases, especially atherosclerotic diseases, are known to be associated with oxidative stress produced by excessive reactive oxygen species (ROS) [1,2]. The sestrin family is comprised of antioxidant proteins, and mammals express three sestrins that share nearly 50% identical amino acid sequences: sestrin1, sestrin2, and sestrin3. Sestrins protect against the oxidative stress that is induced by cell or tissue injury by relieving oxidative stress [3,4,5]. In 1999, sestrin1 was identified as p53-activated gene 26 (PA26), and sestrin2 and sestrin3 were discovered in 2002. Sestrin1 and sestrin2 are recognized to be PA26-related proteins and are regulated mainly by p53 which is induced by oxidative stress, genotoxic stress, and oncogenic stress. Sestrin3 was identified as a PA26 structure-related gene induced by forkhead box O (FOXO) transcription factors, which also contribute to the induction of sestrin1 [3,4,5]. Among the members of the sestrin family, sestrin2 has been most extensively investigated; it has been demonstrated to be an important stress-induced protein that is responsive to various stresses such as hypoxia, DNA damage, and oxidative stress, and sestrin2 is widely expressed in mammals [3,6]. Sestrin2 has a protective effect on physiological and pathological states, mainly by regulating oxidative stress, endoplasmic reticulum stress, autophagy, metabolism, and inflammation [7]. In addition, sestrin2 regulates cell growth, metabolism and survival in response to various stresses. Its anti-oxidative and anti-aging roles have been the focus of many recent studies [8].

This review describes the protective role of sestrin2 against the progression of atherosclerotic and cardiac diseases.

## 2. Physiopathological Mechanisms of Sestrin2

### 2.1. Sestrin2 Signaling Pathways

Sestrin2, a homolog of PA26, is a highly conserved anti-oxidant protein that was originally identified as hypoxia-induced gene 95 (Hi95) [3,4]. Sestrin2 is expressed and secreted mainly by macrophages, T lymphocytes, endothelial cells, and cardiac fibroblasts and cardiomyocytes [6,9,10]. Sestrin2 was shown to accumulate in cells exposed to stress and to play an important role in suppressing the production of ROS, thus protecting cells from oxidative damage [4]. Sestrin2 is recognized to be a negative regulator of mammalian target of rapamycin (mTOR) signaling through its activation of AMP-dependent protein kinase (AMPK) and its phosphorylation of tuberous sclerosis complex 2 (TSC2) [11]. The expression of sestrin2 is regulated by several transcription factors such as p53, activator protein-1 (AP-1), hypoxia inducible factor (HIF)-1α, and nuclear erythroid-related factor 2 (Nrf2), which are activated by stress responses [4,10]. Sestrin2 functions as a stress-inducible metabolic regulator by inhibiting oxidative stress and proinflammatory signaling, mainly via mechanisms that are dependent on AMPK and mTOR complex 1 (mTORC1) [12,13].

Sestrin2 has been recognized to play a protective role against oxidative stress, mainly via the two signaling pathways of Kelch-like ECH-associated protein 1 (Keap1)/Nrf2 and AMPK/mTORC1 as follows:

Keap1 is a cysteine-rich protein that represses Nrf2 activity. Nrf2 localizes in the cytoplasm and binds to Keap1. The Keap1/Nrf2 signaling plays a critical role in maintaining cell homeostasis under oxidative stress. Sestrin2, activated by oxidative stress, increases the expression of sulfiredoxin through the activation of Nrf2. Sestrin2 can also activate Nrf2 by promoting a p62-dependent autophagic degradation of Keap1. Sestrin2 thus decreases the accumulation of ROS and stimulates anti-oxidant defenses via Keap1/Nrf2 signaling activation [8,14].

TOR is a pivotal regulator of cell growth, proliferation, metabolism and autophagy. TOR was first identified as a protein kinase inhibited by rapamycin. mTOR is present in two distinct complexes, mTORC1, which is sensitive to rapamycin, and mTORC2, which is not [15,16]. The regulation of mTORC1 is dependent on tuberous sclerosis complex 1 and 2 (TSC1 and TSC2), of which TSC2 acts as a GTPase-activating protein for the small GTPase Rheb, which negatively controls Rheb and activates mTOR [15,16]. AMPK is an important nutrient-sensing protein kinase that plays a critical role in maintaining metabolic homeostasis [8,17]. The mechanisms of AMPK activation include phosphorylation by its upstream kinases, such as liver kinase B1 (LKB1). Sestrin2 induced by stress inhibits mTORC1 through AMPK activation. Moreover, mTORC1 inhibits autophagy, which plays a critical role in cell viability [18]. As an inhibitor of mTORC1, sestrin2 promotes autophagy. The AMPK/mTORC1 signaling pathway is known to be critical for the role of sestrin2 in controlling cell metabolism and survival under stress conditions [8].

**Figure 1:**
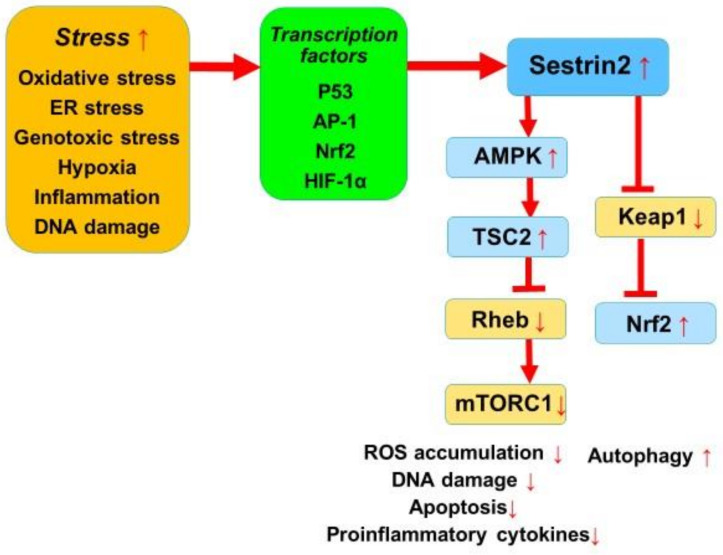
A summary of the main signaling pathways and pathophysiological mechanisms of sestrin2.

The main signaling pathways and pathophysiological mechanisms of sestrin2 are summarized in Figure 1.

### 2.2. Sestrin2 and Oxidative Stress

Sestrin2 acts as an anti-oxidant protein that diminishes the accumulation of ROS and inhibits mTORC1 signaling. Both the accumulation of ROS and the activation of mTORC1 were shown to be associated with aging and age-related diseases, such as atherosclerotic disease [19].

Hu et al. showed that the expression of sestrin2 was upregulated in macrophages by oxidized low-density lipoprotein (LDL) in a time-dependent and dose-dependent manner, and that the knockdown of sestrin2 with small RNA interference promoted cell apoptosis and the production of ROS induced by oxidized LDL [9]. They suggested that the induction of sestrin2 acts as a compensatory response to oxidized LDL for cell survival.

Yang et al. also reported that the downregulation of sestrin2 increased the production of ROS and elevated blood pressure in mice with dopamine D2 receptor (D2R) silent-induced hypertension [20]. D2R decreased the renal production of ROS and regulated blood pressure, in part via the positive regulation of paraoxonase 2. In D2R-deficient mice, the silencing of D2R in renal proximal tubule cells decreased the expression of sestrin2 and increased hyperoxidized peroxiredoxins. In contrast, D2R stimulation in renal proximal tubule cells increased the sestrin2 expression, decreased hyperoxidized peroxiredoxins, and reduced ROS production. Silencing sestrin2 in renal proximal tubule cells increased hyperoxidized peroxiredoxins and ROS production, abolished a D2R-induced decrease in peroxiredoxin hyperoxidation, and prevented the inhibitory effect of D2R stimulation on ROS production. In mice, renal selective silencing of sestrin2 by small interfering RNA increased renal oxidative stress and blood pressure values. These findings thus suggest that D2R, via paraoxonase 2 and sestrin2, maintains the normal renal redox balance, which contributes to the maintenance of normal blood pressure.

In dendritic cells, sestrin2 knockdown was shown to promote endoplasmic reticulum (ER) stress-related apoptosis and to exacerbate ER disruption and the formation of dilated and aggregated structures [21]. The overexpression of sestrin2 in dendritic cells markedly decreased the apoptotic rate and inhibited ER stress-related protein translation. Moreover, sestrin-deficient mice demonstrated increased apoptosis and an aggravated extent of ER stress. These findings suggest that sestrin2 is a potential regulator to inhibit ER stress signaling that exerts a protective effect on apoptosis in dendritic cells.

The accumulating evidence suggests that sestrin2 acts as an anti-oxidant protein and reduces the production and accumulation of ROS.

### 2.3. Sestrin2 and Inflammation

Atherosclerotic disease such as coronary artery disease (CAD) is recognized to be a chronic inflammatory disease as well as an age-related disease, and oxidative stress produced by excessive ROS contributes to the pathogenesis of atherosclerosis [1,22]. The excessive production of ROS is implicated in vascular injury. Endogenous anti-oxidants are thought to play a protective role against the progression of atherosclerosis, and the imbalance in oxidant and anti-oxidant mechanisms leads to a state of oxidative stress [1,22]. Atherosclerotic disease is characterized as involving an imbalance between the formation of ROS and the ROS-degrading antioxidant system [23]. Since the progression of atherosclerosis is associated with inflammation with oxidative stress [1,22], sestrin2 would play a protective role against atherosclerotic disease [8,12].

Kim et al. reported that lipopolysaccharide (LPS), a Toll-like receptor 4 (TLR4) ligand, increased the levels of sestrin2 mRNA and protein in macrophages, whereas sestrin1 mRNA and protein were not affected by LPS [10]. The same researchers also showed that sestrin2 almost completely inhibited an LPS-induced release of nitric oxide (NO) and the expression of inducible nitric oxide synthase (iNOS) [24]. Their sestrin2 knockout experiment confirmed the role of sestrin2 in LPS-activated macrophages. The release and expression of proinflammatory cytokines such as tumor necrosis factor (TNF)-α, interleukin (IL)-6 and IL-1β were shown to be inhibited in sestrin2-expressing cells. Sestrin2 prevented LPS-induced apoptosis and ROS production via the inhibition of NADPH oxidase. Those authors suggested that sestrin2 inhibits TLR-induced proinflammatory signaling and protects cells by inhibiting c-Jun N-terminal kinase (JNK)- or p38-mediated c-Jun phosphorylation [24].

Hwang et al. demonstrated that the knockdown of sestrin2 in human umbilical vein endothelial cells (HUVECs) promoted LPS-induced inflammatory responses, apoptosis, and ROS production [25]. In sestrin2-knockdown HUVECs, the LPS-mediated nuclear factor kappa Β (NF-κB) phosphorylation, the secretion of pro-inflammatory cytokines such as TNF-α, monocyte chemotactic protein-1 (MCP-1), and IL-6, and the expression of adhesion molecules were significantly increased. LPS-induced ROS production, ER stress, and cell toxicity were also increased after sestrin2 knockdown. All of these effects were fully abrogated by treatment with an AMPK activator. Treatment with an AMPK activator was shown to reduce atherosclerotic lesions in apolipoprotein E-knockout mice [26], and in the aortic tissue samples from the mice, sestrin2 knockdown resulted in the reduction in AMPK phosphorylation and the induction of LPS-mediated NF-κB phosphorylation, thereby leading to the up-regulation of adhesion molecules and ER stress-related signaling [25]. These findings thus suggested that sestrin2 knockdown aggravates atherosclerotic processes by increasing pro-inflammatory reactions and ER stress in the endothelium via an AMPK-dependent mechanism. 

These findings therefore indicate that sestrin2 has anti-oxidant and anti-inflammatory properties, thereby protecting against the progression of atherosclerosis. Sestrin2 might be a beneficial target for the amelioration of atherosclerosis, indicating its potential as a therapeutic target in atherosclerotic diseases.

Angiotensin II (AngII) are recognized to induce apoptosis via the activation of AngII receptors, and the activation of AngII signaling triggers proinflammatory effects and the production of ROS in vascular walls by inducing multiple downstream pathways, which consequently causes endothelial dysfunction and cardiovascular diseases [27]. Yi et al. showed that AngII induced the expression of sestrin2 in HUVECs in a time-dependent and dose-dependent manner and that the knockdown of sestrin2 using small RNA interference promoted the cellular toxicity of AngII as well as the reduction in cell viability, the exacerbation of oxidative stress, and the stimulation of apoptosis [28]. They indicated that sestrin2 induction acts as a compensatory response to AngII for survival, implying that the stimulation of sestrin2 expression might be an effective pharmacological target for the treatment of AngII-associated cardiovascular diseases.

In the early phase of atherosclerosis, macrophage-derived foam cells play a major role in atherosclerosis. The AMPK and mTOR signaling pathways were reported to play an essential role in the initiation and progression of atherosclerosis [29,30], and sestrin2 was shown to modulate mTOR activity, thereby regulating glucose and lipid metabolism [31,32]. Sundararajan et al. reported that high-glucose and oxidized LDL treatment mediated the production of proinflammatory cytokine (M1) with a concomitant decrease in anti-inflammatory cytokine (M2) levels in macrophages [33]. M2 macrophages promote the secretion of IL-10, transforming growth factor-β (TGF-β), and extracellular matrix to protect vessels from atherosclerosis, whereas M1 macrophages secrete matrix metalloproteinases (MMPs) and proinflammatory factors such as TNF-α, IL-6, and IL-1β [34]. Glucose and oxidized LDL increased mTOR activation with a marked reduction in AMPK and sestrin2 expression and increased foam cell formation and monocyte adhesion to endothelial cells [33]. In addition, the overexpression of sestrin2 regulated the polarization of macrophages toward anti-inflammatory M2, while the knockdown of sestrin2 directed the polarization toward proinflammatory M1 [33]. These findings suggested that sestrin2 plays a major role in regulating monocyte activation via the AMPK-mTOR signaling pathway under diabetic and dyslipidemic conditions and that sestrin2 has a protective role against atherosclerosis.

## 3. Sestrin2 and Cardiac Diseases

### 3.1. Aging and Myocardial Infarction

The hallmarks of aging include accumulated aberrant proteins and oxidative stress, dysfunction of cellular metabolism and organs, and defective homeostasis. It was shown that AMPK activation, mTORC1 suppression, and autophagy stimulation are beneficial for extending lifespan [35]. Sestrin2 is suggested to be associated with aging and age-related diseases [12,36], and blood sestrin2 levels were reported to correlate with age [37,38]. Kishimoto et al. also reported that plasma sestrin2 levels correlated with age (r = 0.29) in 304 patients undergoing coronary angiography [39]. Moreover, sestrin2 was demonstrated to be associated with an aging-related process, and physical exercise can upregulate sestrin2 [40]. Sestrin2 is abundantly expressed in skeletal muscles and is important to the maintenance of muscle homeostasis. Rai et al. reported that the serum sestrin2 levels in 51 frail elderly subjects were low [41]. These findings suggest that sestrin2 is associated with frailty in the elderly.

Damaged myocardium from an ischemia reperfusion (I/R) injury initiates inflammatory responses including those involving macrophages, neutrophils, and inflammatory cytokines [42]. Sestrin2 is suggested to be cardioprotective against inflammation, oxidative stress, and I/R stress. Morrison et al. reported that sestrin2 knockout mice had greater-than-normal myocardial infarct sizes and impaired cardiac function with impaired AMPK activation, and sestrin2 was observed to promote AMPK activation during ischemia and to initiate AMPK phosphorylation via an interaction with LKB1. These findings suggest that sestrin2 plays an important role in cardiac protection against I/R injury, serving as an LKB1-AMPK scaffold to initiate the activation of AMPK during ischemia [43]. The same research group also showed that sestrin2 is an age-related protein that decreases with aging and contributes to the susceptibility of the aged heart toward I/R injury [44]. In mice, ischemic AMPK activation was blunted, and the infarct size was larger in the aged hearts [44]. Moreover, an adeno-associated virus delivery of sestrin2 significantly rescued sestrin2 protein level and ischemic tolerance of aged hearts. These findings suggest that (1) sestrin2 is a scaffold protein that mediates the activation of AMPK in ischemic myocardium, and (2) decreased sestrin2 levels in aging led to blunted ischemic AMPK activation and increased the sensitivity to ischemic insults. In addition, the sestrin2 knockout mice showed an aged-like phenotype in their hearts with excessive oxidative stress, disorganized myocardium, and transcriptomic alterations with I/R stress similar to aged mice [45]. Sestrin2 deficiency increased oxidative stress with up-regulated proinflammatory signaling and greater myocardial damage after I/R stress. These findings indicate that sestrin2 plays critical roles in modulating inflammation and apoptosis in the murine hearts in response to I/R injury.

The inflammatory response plays a crucial role in the progress of myocardial infarction (MI), where it is important in the repair of the myocardium but is also involved in myocardial remodeling and heart failure after an MI. The post-MI inflammatory response includes a substantial recruitment of circulating leukocytes into the infarcted myocardium. Among these infiltrating leukocytes, macrophages are critical players in the modulation of the post-MI inflammatory response [46]. The sestrin2 expression in cardiac macrophages was shown to be upregulated in a mouse model of MI [47]. Sestrin2 overexpression suppressed the inflammatory response of M1 macrophages through the inhibition of mTORC1 signaling both in vitro and in vivo. Together these findings suggest that sestrin2 plays a role in the post-MI inflammatory response, thereby leading to myocardial repair and remodeling.

### 3.2. Cardiomyopathy

In sestrin2 knockout mice, significantly reduced cardiac function with increased myocardial fibrosis was demonstrated after irradiation [48]. This suggests that sestrin2 is involved in the development of cardiomyopathy after irradiation. Li et al. also reported that the sestrin2 expression was significantly increased after an injection of doxorubicin in mice [49]. In sestrin2 knockout mice, sestrin2 deficiency rendered the mice more vulnerable to doxorubicin and exacerbated doxorubicin-induced cardiomyocyte apoptosis and cardiac dysfunction. Li et al. suggested that sestrin2 is a cardioprotective protein that minimizes cardiac damage in response to doxorubicin insult.

In rat cardiomyocytes, sestrin2 knockdown reduced the AMPK phosphorylation, downregulated antioxidant genes, and increased the ROS production upon LPS treatment [50]. LPS-mediated apoptosis and the expression of MMPs were significantly increased, and these increases were prevented by treatment with 5-aminoimidazole-4-carboxamide ribonucleotide (AICAR), an AMPK activator. Moreover, AMPK phosphorylation was shown to be decreased in the heart tissue from sestrin2 knockdown mice, which was associated with decreased antioxidant gene expression and increased apoptosis. Decreased AMPK phosphorylation by sestrin2 knockdown increased the LPS-mediated expression of cardiac fibrotic factors (e.g., collagen type I and type III). These results suggest that sestrin2 may play a role in the development of cardiomyopathy under inflammatory conditions.

In rats, sestrin2 knockdown was reported to aggravate the cardiomyocyte hypertrophy induced by phenylephrine, and sestrin2 overexpression protected cardiomyocytes from phenylephrine-induced hypertrophy, suggesting a protective effect of sestrin2 against cardiomyocyte hypertrophy [36]. In addition, Quan et al. reported that sestrin2 knockout mice had larger hearts and impaired cardiac function after aortic constriction for pressure overload [51]. Hypertension is the main pathological factor in the development of heart failure, and heart failure caused by hypertension is characterized by cardiac hypertrophy. An adeno-associated virus delivery of sestrin2 rescued the sestrin2 expression, attenuated the activation of mTORC1 and increased the pressure overload tolerance in hearts [51]. These results indicate that sestrin2 inhibits myocardial hypertrophy by inhibiting the mTORC1 pathway, thereby regulating protein synthesis, metabolism, autophagy, and apoptosis.

Wang et al. investigated the plasma sestrin2 levels in 220 patients with heart failure and reported that the patients’ plasma sestrin2 levels were high and that their sestrin2 levels gradually increased as the heart failure progressed from New York Heart Association (NYHA) functional class II to IV [6]. High sestrin2 levels were also shown to be associated with major adverse cardiac events in the patients with heart failure. Thus, high plasma levels of sestrin2 in patients with heart failure may reflect a compensatory response to the heart failure and may help protect against the development of adverse cardiac events. However, the main source and role of high plasma sestrin2 levels in patients with heart failure remain unclear.

The accumulating evidence suggests a role for sestrin2 in the development and progression of cardiomyopathy and heart failure. However, further studies in humans are needed to clarify the roles of sestrin2 in cardiomyopathy and heart failure.

## 4. Sestrin2 and Atherosclerotic Diseases

### 4.1. Coronary Artery Disease

Regarding the association between sestrin2 levels and coronary atherosclerosis, Ye et al. measured the plasma sestrin2 levels in 114 patients with CAD (stable angina (SA), *n* = 44; unstable angina (UA), *n* = 41; acute MI (AMI), *n* = 29) and 35 without CAD [37]. They reported that the sestrin2 levels were higher in the patients with CAD than in those without CAD and that the levels were much higher in the patients with UA or AMI compared to the patients with SA. However, a multivariate analysis was not performed in that study despite the involvement of some significant differences in atherosclerotic risk factors between the patients with and without CAD. Ye et al. also showed that the sestrin2 levels correlated with the Gensini score (r = 0.46), but this correlation coefficient was analyzed in all patients with CAD including those with AMI.

**Figure 2:**
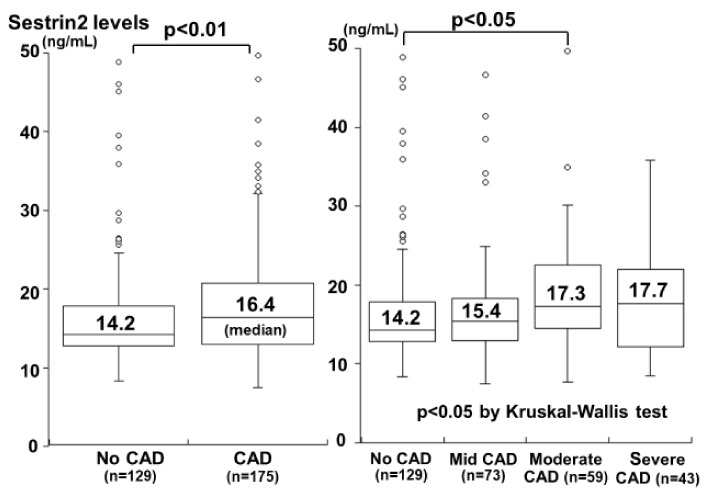
Plasma sestrin2 levels and the presence and severity of CAD. Plasma sestrin2 levels were significantly higher in patients with CAD than in those without CAD (**left**). The sestrin2 levels increased in a stepwise manner depending on the severity of CAD (defined as the number of >50% stenotic vessels) and were highest in the severe CAD group (**right**). The central lines represent the median, and the boxes represent the 25th to 75th percentiles. The whiskers represent the lowest and highest values in the 25th percentile minus 1.5 interquartile range (IQR) and the 75th percentile plus 1.5 IQR, respectively (modified from Kishimoto et al. [39]).

Kishimoto et al. recently investigated plasma sestrin2 levels in 304 patients undergoing coronary angiography for suspected CAD [39]. Patients with acute coronary syndrome, defined as acute MI and UA, were excluded from the study, and since plasma sestrin2 levels in patients with heart failure were reported to be high [6], patients with heart failure were also excluded. Notably, the plasma sestrin2 levels were significantly higher in the patients with CAD than in those without CAD (Figure 2). A stepwise increase in sestrin2 levels was also observed that depended on the severity of CAD (defined as the number of >50% stenotic vessels), and the sestrin2 levels were highest in the patients with severe CAD (Figure 2). The sestrin2 levels were significantly but weakly correlated with the number of >50% stenotic segments (r = 0.12) and age (r = 0.29). In a multivariate analysis, the plasma sestrin2 level was a significant factor associated with CAD independent of atherosclerotic risk factors, and the odds ratio for CAD was 1.79 (95%CI: 1.09–2.95) for a high sestrin2 level (>16.0 ng/mL). Thus, high plasma sestrin2 levels were identified in the patients with CAD. However, because sestrin2 is secreted by various types of cells including macrophages, T lymphocytes, endothelial cells, and cardiomyocytes [6,9,10] and because sestrin2 levels were not measured in the coronary sinus in the Kishimoto et al. study [39], the main sources of sestrin2 in patients with CAD remain unclear. Moreover, as shown in Figure 2, there was a substantial overlap in sestrin2 levels between the patients with and without CAD. Therefore, plasma sestrin2 levels in patients with CAD may reflect not only the degree of coronary atherosclerosis but also the degree of atherosclerosis in other vascular beds. Since sestrin2 is considered to have anti-oxidant and anti-inflammatory properties, high plasma levels of sestrin2 in patients with CAD may reflect a compensatory response to increased oxidative stress and may contribute to protection against the progression of CAD. However, further studies are needed to elucidate the main source and role of high plasma sestrin2 levels in patients with CAD. 

### 4.2. Carotid Atherosclerosis

Nutritional abundance can lead to chronic mTORC activation, thereby enhancing protein and lipid biosynthesis and inhibiting autophagic catabolism [52]. Chronic mTORC1 stimulation with autophagy inhibition in hepatocytes was reported to lead to insulin resistance and type 2 diabetes mellitus (DM) mainly via the inhibition of phosphorylation of insulin receptor substrates [32]. In obese sestrin2-knockout mice, sestrin2 deficiency was shown to exacerbate obesity-induced mTORC activation, glucose intolerance, insulin resistance, and hepatosteatosis, all of which were reversed by AMPK activation [31]. These findings suggest that sestrin2 may play a role in glucose metabolism. Since DM is a well-known atherosclerotic risk factors, sestrin2 may be associated with an increased risk of atherosclerotic diseases.

Sundararajan et al. reported that the serum sestrin2 levels in 81 patients with type 2 DM were significantly lower compared to those of 46 subjects with normal glucose tolerance [53]. In contrast, Chung et al. investigated the serum sestrin2 levels and the carotid intima-media thickness (IMT) measured by ultrasonography in 46 subjects without DM and 194 with DM [38]; they reported that the sestrin2 levels did not differ among the normal subjects, the DM patients without carotid atherosclerosis, and the DM patients with carotid atherosclerosis. No significant correlation was detected between the sestrin2 levels and carotid IMT, but the sestrin2 levels were correlated with the homeostatic model assessment of insulin resistance (HOMA-IR), waist circumference, percentage body fat, and truncal fat mass. 

**Figure 3:**
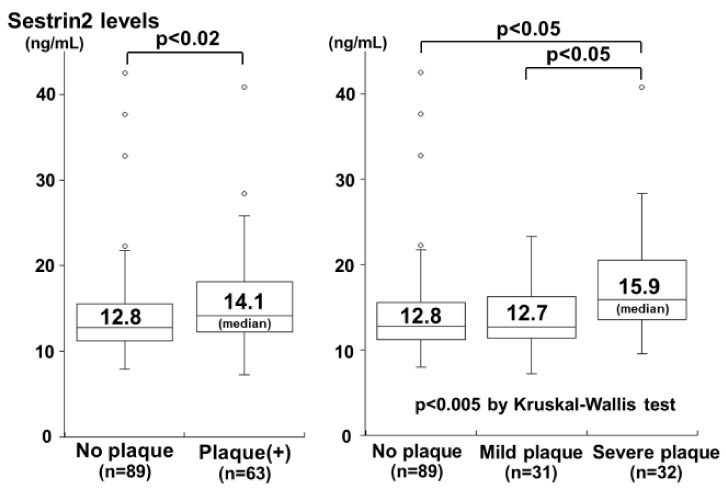
Plasma sestrin2 levels and the presence and severity of carotid plaque. Plasma sestrin2 levels were significantly higher in subjects with plaque than in those without plaque (**left**). Sestrin2 levels increased in a stepwise manner depending on the severity of plaque and were highest in the subjects with severe plaque (score ≥ 2) (**right**) (modified from Kishimoto et al. [54] copyright (2020), with permission from Elsevier).

Kishimoto et al. investigated the plasma sestrin2 levels in 152 subjects (mean age 65 ± 10 years) undergoing carotid ultrasonography for a medical check-up for an evaluation of atherosclerosis [54]. No significant correlation between sestrin2 levels and carotid IMT was identified; however, the plasma sestrin2 levels were significantly higher in the subjects with carotid plaque than in those without plaque (Figure 3). In addition, the sestrin2 levels increased in a stepwise manner depending on the severity of plaque (defined as the plaque score) and were highest in the subjects with severe plaques (Figure 3). Moreover, the plasma sestrin2 levels were significantly correlated with the plaque score (r = 0.24). In a multivariate analysis, age and male gender were significant factors for the presence of plaque, but the sestrin2 level was not. However, the sestrin2 level was a significant factor for severe plaque (plaque score ≥2) independent of atherosclerotic risk factors. The odds ratio for severe plaque was 5.70 (95%CI: 1.99–16.35) for a high sestrin2 level (>13.0 ng/mL). These findings demonstrated that the plasma sestrin2 levels were high in individuals with carotid plaques and were associated with the severity of carotid atherosclerosis, as reported in patients with CAD [39]. High plasma sestrin2 levels may reflect an increased oxidative stress condition and may function to help protect the body against the progression of atherosclerosis.

### 4.3. Miscellaneous

In addition to CAD and carotid atherosclerosis, Xiao et al. reported that the sestrin2 expression was upregulated in the aortic specimens from 12 patients with aortic dissection, which is recognized to be related to atherosclerosis, and that the plasma sestrin2 levels were high in 120 patients with aortic dissection [55]. They suggested that the upregulation of sestrin2 may help reduce ROS, inflammation, and apoptosis in the aortic tissues of patients with aortic dissection. However, it has not been determined whether or not high plasma sestrin2 levels have a preventative effect on the development of aortic dissection.

Obstructive sleep apnea (OSA) is known to be common among obese individuals. OSA is characterized by repeated apnea during sleep and by intermittent hypoxia, leading to serious complications such as hypertension, DM, and CAD, due to oxidative stress. Intermittent hypoxia increases HIF-1α, thereby leading to an increase in sestrin2 expression. Jiang et al. reported that plasma sestrin2 levels were high in 36 patients with OSA, and that sestrin2 levels decreased after 4-week continuous positive airway pressure (CPAP) treatment [56]. They suggested that high plasma sestrin2 levels may contribute to a reduction in complications such as CAD as well as oxidative stress. 

The blood sestrin2 levels in atherosclerotic and cardiometabolic diseases are summarized in Table 1.

## 5. Conclusions

The above results support the notion that sestrin2 has anti-oxidant and anti-inflammatory properties and that sestrin2 may play a protective role against the progression of atherosclerotic diseases such as CAD and carotid atherosclerosis. Sestrin2 may thus be a potential therapeutic target in atherosclerotic diseases. However, since the plasma sestrin2 levels in patients with CAD and those with carotid atherosclerosis were shown to be high, it remains unclear whether or not an exogenous administration of sestrin2 could be beneficial for the prevention of atherosclerotic disease. Further studies are needed to elucidate the roles of high plasma sestrin2 levels in patients with atherosclerotic diseases.

## Figures and Tables

**Table 1:** Summary of blood sestrin2 levels in patients with and without atherosclerotic and cardiometabolic diseases.

Study	Serum/Plasma	Study Subjects	Results
Rai et al. [41]	Serum	51 frail elderly vs. 41 non-frail elderly	Lower in frail elderly than in non-frail elderly.
Wang et al. [6]	Plasma	220 patients with HF vs. 80 controls	Higher in patients with HF than in controls
Xiao et al. [55]	Plasma	120 patients with aortic dissection vs. 40 without dissection	Higher in patients with aortic dissection than in those without dissection
Jiang et al. [56]	Plasma	36 patients with OSA vs. 21 controls	Higher in patients with OSA than in controls
Ye et al. [37]	Plasma	114 patients with CAD (44 SA, 41 UA, 29 AMI) vs. 35 without CAD	Higher in patients with CAD than in those without CAD Higher in patients with UA and AMI than in those with SA
Kishimoto et al. [39]	Plasma	175 patients with CAD vs. 129 without CAD	Higher in patients with CAD than in those without CAD
Sundararajan et al. [53]	Serum	81 patients with DM vs. 46 controls (NGT)	Lower in patients with DM than in controls
Chung et al. [38]	Serum	194 patients with DM vs. 46 without DM	No difference between patients with and without DM
Chung et al. [38]	Serum	80 DM patients with carotid atherosclerosis vs. 114 DM without carotid atherosclerosis	No difference between DM patients with and without carotid atherosclerosis
Kishimoto et al. [54]	Plasma	63 subjects with carotid plaque vs. 89 without carotid plaque	Higher in subjects with carotid plaque than in those without carotid plaque
